# Supplementary material for: Concerted Regulation of Glycosylation Factors Sustains Tissue Identity and Function
Source: Biomedicines. 2022 Jul 27;10(8):1805. doi: 10.3390/biomedicines10081805 (PMC9404854; doi:10.3390/biomedicines10081805)
Supplement: Supplementary file 1 [file biomedicines-10-01805-s001.zip › SuppFigures.pdf]

Supplementary Figure S1

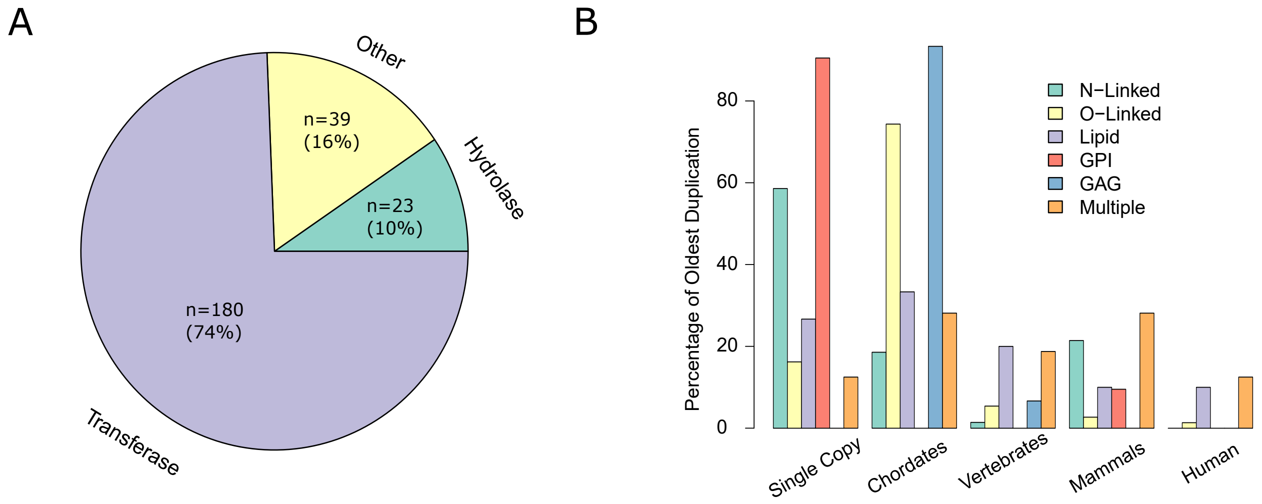

Supplementary Figure S1: Glycosylation Factors are an evolutionarily conserved family of genes. **A)** Pie chart with the frequencies of each glycosylation enzymatic subclass among the 242 genes considered in this study. The category “Other” includes transporters, isomerases, and enzymes involved in the GPI anchor. **B)** Barplot with the percentages of the oldest phylum where a paralogous gene copy occurred, for each glycosylation subclass.

Supplementary Figure S2

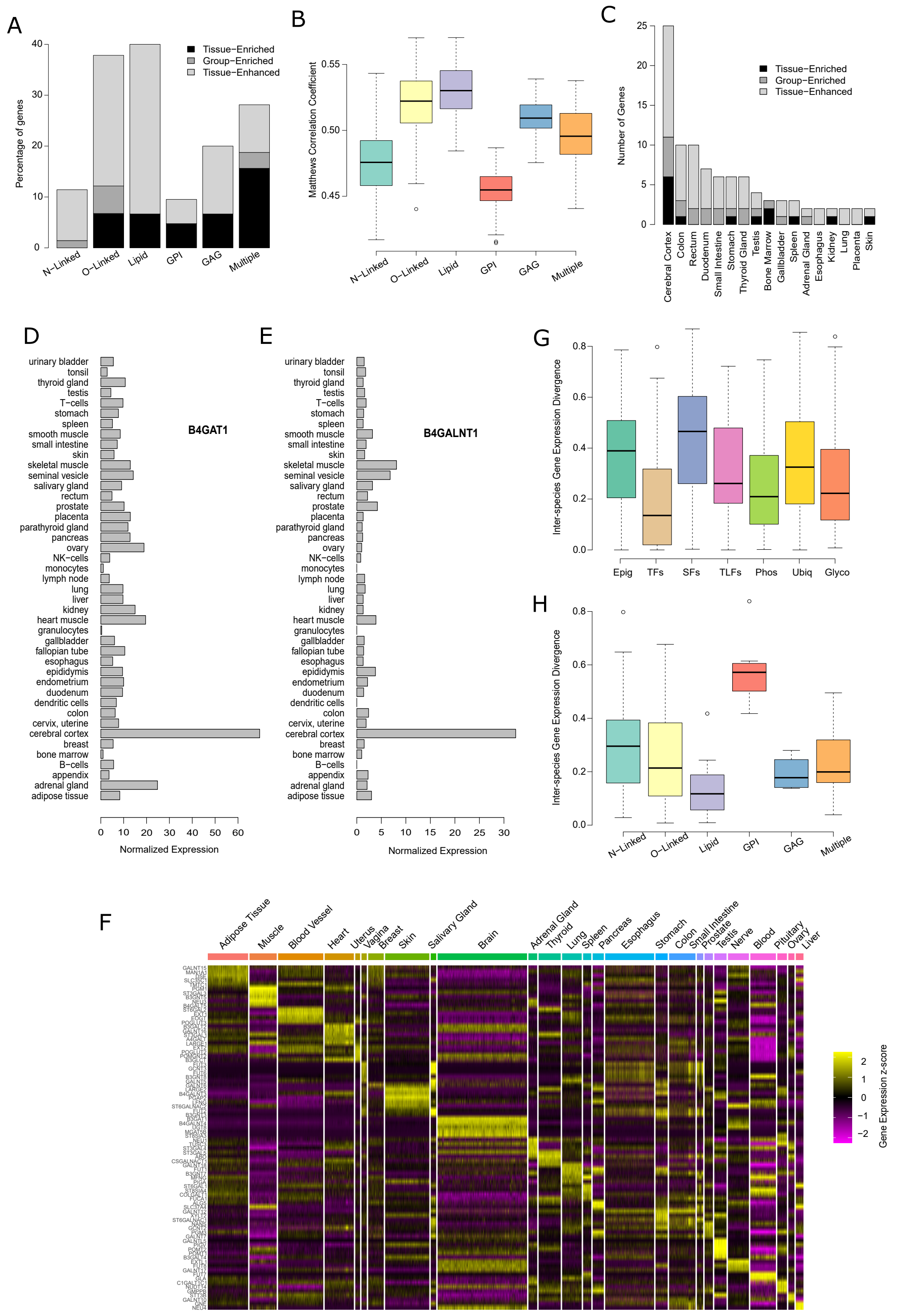

Supplementary Figure S2: Glycosylation Factors show tissue-specific expression. **A)** Cumulative barplot representation of the frequency of genes belonging to each glycosylation subclass that show tissue-specific expressionin the protein atlas (colors represent degree of specificity, from the most specific Tissue-Enriched to the least specific Tissue-Enhanced). **B)** Boxplot representation of the Matthews Correlation Coefficient (MCC) value for the classification of the tissue of each GTEX sample (n=100 random forest models of 30 randomly selected genes of each glycosylation subclass). **C)** Cumulative barplot representation of the number of genes that show tissue-specific expression in the protein atlas, within a given tissue (colors represent degree of specificity, from the most specific Tissue-Enriched to the least specific Tissue-Enhanced). **D)** Barplot representation of the consensus normalized expression ("NX") value of the gene B4GAT1, for the different tissues in protein atlas. **E)** Barplot representation of the NX value of the gene B4GALNT1, for the different tissues in protein atlas. **F)** Heatmap representation of the top5 glycosylation factors that are markers of GTEX tissues (with greatest fold change between average expression in the tissue against the other tissues, and adjusted p-valueof a wilcoxon test < 0.05). Only tissues with more than 100 samples were considered. Some genes may be markers of more than one tissue. **G)** Boxplot representation of the within-tissue normalized standard deviation of orthologous gene expression (from Brawand et al. 2011) in the tissues, for each gene of the different classes of genes. **H)** Boxplot representation of the within-tissue normalized standard deviation of orthologous gene expression(from Brawand et al. 2011) in the tissues, for each gene of the different glycosylation subclasses.

Supplementary Figure S3

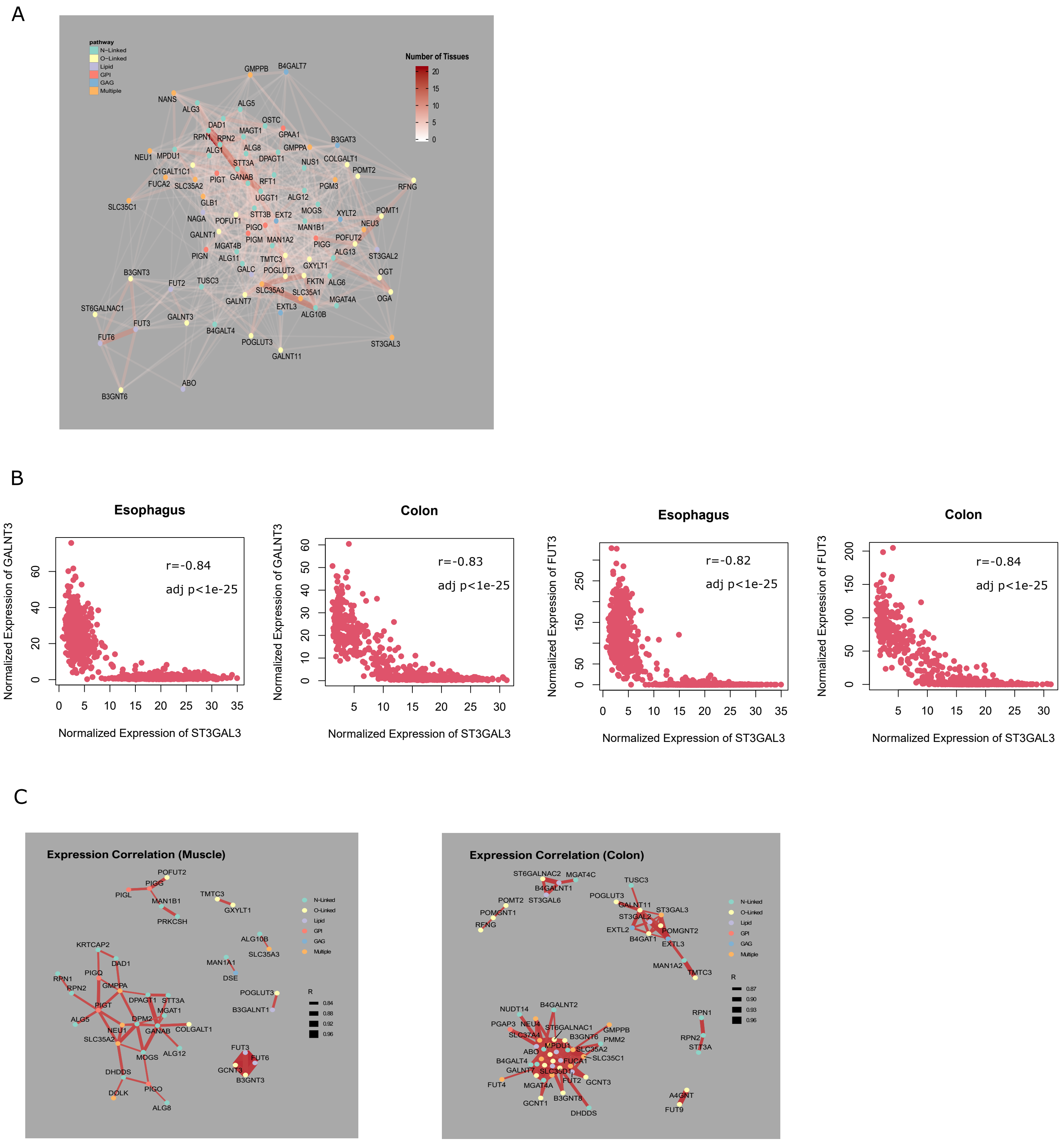

Supplementary Figue S3: Glycosylation Factors seem to be organized in clusters of co-expression. **A)** Network representation of the number of tissues with high positive correlation between pairs of glycosylation factors (based on the gene expression of GTEX samples, R>0, adjusted p < 0.05). Node colors represent glycosylation subclass, color intensity and line thickness represent the number of tissues where the gene pair displays a high degree of correlation. **B)** Pairwise comparison of normalized expression of pairs of glycosylation factors in different tissues. Each dot represents one sample. **C)** Network representation of highly correlated pairs of glycosylation factors (based on the gene expression of GTEX samples, R>0.8, ajusted p < 0.05) in muscle and colon samples. Node colors represent glycosylation subclass, line thickness represent degree of correlation.

Supplementary Figure S4

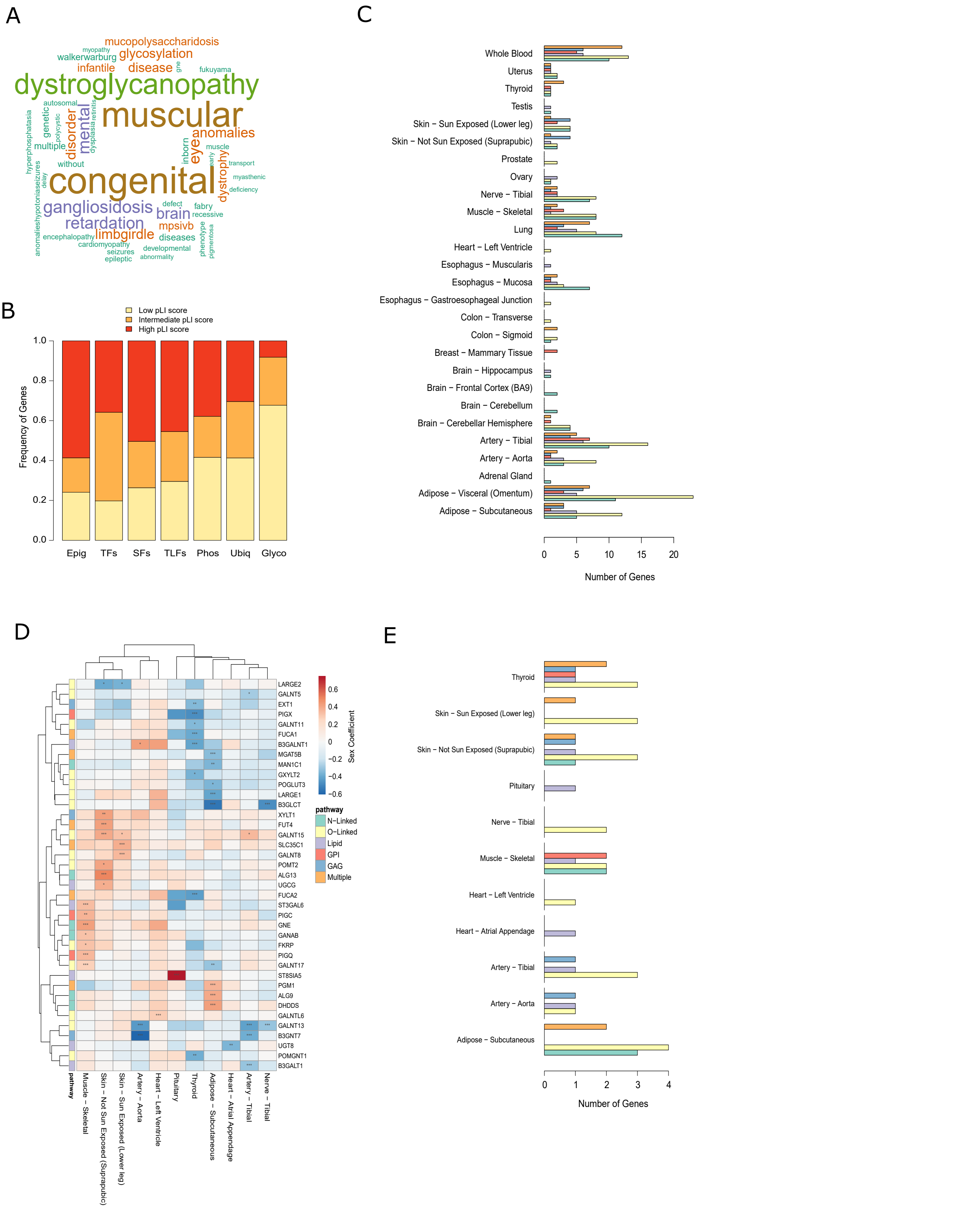

Supplementary Figure S4: Glycosylation Factors are clinically relevant. **A)** Wordcloud representation of the frequency of terms associated with glycosylation factors in the Clinvar database. **B)** Stacked bar plot representation of the proportion of genes, within a given gene class, that fall in different categories of pLi score: low (pLI<0.1, light yellow); medium (0.9>=pLI>=0.1, orange); high (pLi>0.9, dark red).

C) Barplot of the number of genes, divided by glycosylation subclass, with significant (p<0.05) age-associated coefficient in a linear model associating age with gene expression in GTEX samples, in a given tissue.

D) Heatmap representation of sex-associated coefficients of glycosylation factors with at least one significant sex-associated coefficient in a linear model associating sex with gene expression in GTEX samples, in a given tissue (*: p<0.05; **: p<0.01; ***: p<0.001). **E)** Bar plot of the number of genes, divided by glycosylation subclass, with significant (p<0.05) sex-associated coefficient in a linear model associating sex with gene expression in GTEX samples, in a given tissue.

Supplementary Figure S5

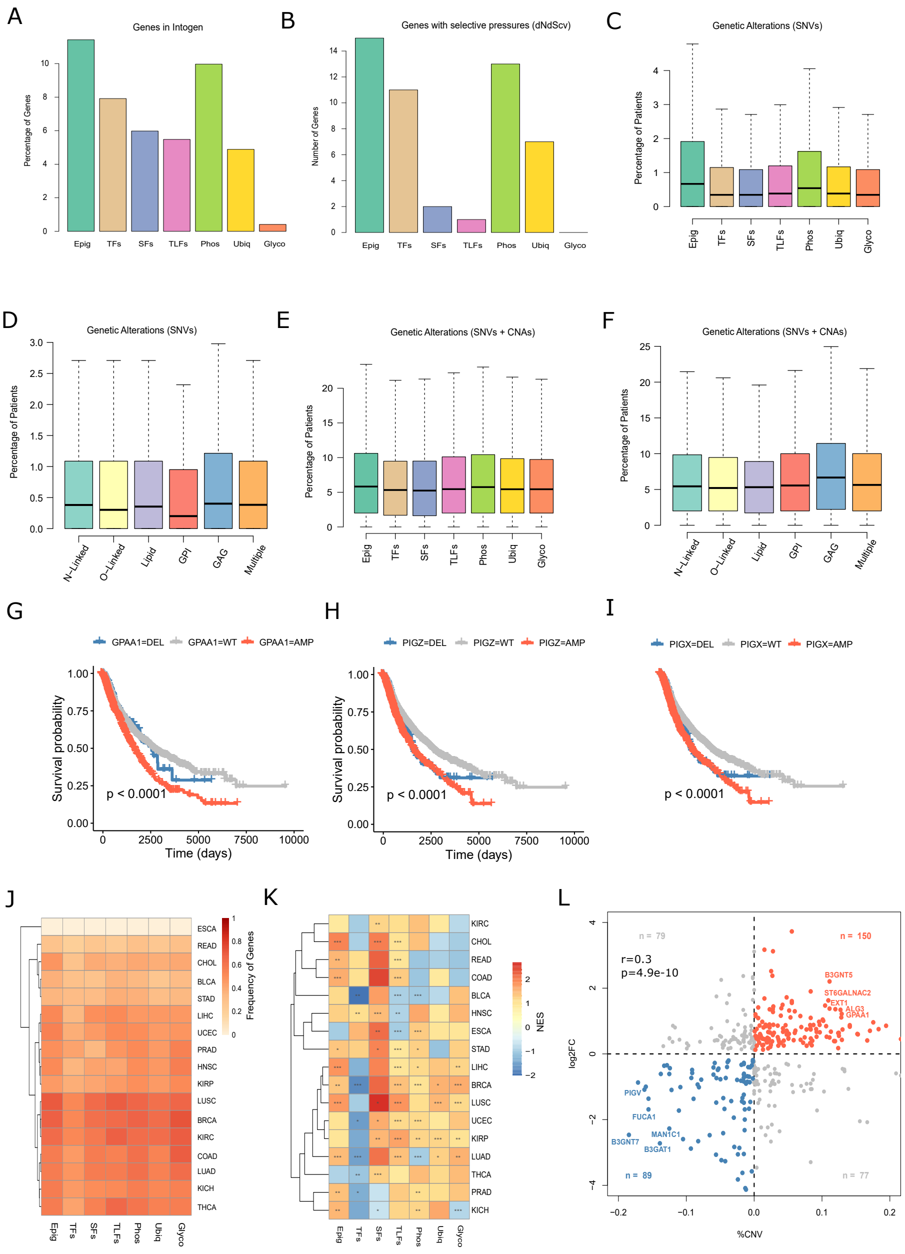

Supplementary Figure S5: Glycosylation Factors are altered in cancer. **A)** Barplot of the frequency of genes of different classes regarding their presence in the intogen database, as having mutations known to be associated with tumor development. **B)** Barplot of the frequency of genes of different classes for which there is evidence, using the dNdScv method, of evolutionary pressure regarding the frequency and types of mutations in TCGA samples. **C)** Boxplot of the per-gene frequency of samples (11124 TCGA samples corresponding to 33 tumor types) harboring tumor-specific mutations (SNPs) in each gene, divided by gene class. **D)** Boxplot of the per-gene frequency of samples (11124 TCGA samples corresponding to 33 tumor types) harboring tumor-specific genetic alterations (SNPs) in each gene, divided by glycosylation subclass. **E)** Boxplot of the per-gene frequency of (over 11124 TCGA samples corresponding to 33 tumor types) harboring tumor-specific mutations (CNAs + SNPs) in each gene, samples divided by gene class **F)** Boxplot of the per-gene frequency of samples (over 11124 TCGA samples corresponding to 33 tumor types) harboring tumor-specific genetic alterations (CNAs + SNPs) in each gene, divided by glycosylation subclass. **G-I)** Kaplan-Meier curves representing the effect on survival time of CNAs in the GPAA1 **(G)**, PIGZ **(H)** and PIGX **(I)** genes (over all TCGA samples, n=9569). Notice that PIGX and PIGZ are closeby in the genome, and that alterations occur in about 15% of patients for the three genes. **J)** Heatmap representation of the frequency of genes, according to their gene class, whose expression is misregulated in cancer (tumor compared to normal from TCGA). **K)** Heatmap representation of the normalized enrichment scores, obtained from a gene set enrichment analysis, of different gene classes in different TCGA cancers (*: $p<0.05$; **: $p<0.01$; ***: $p<0.001$). **L)** Plot representing the log2FC and frequency of copy number alteration events (positive if amplifications are predominant, negative if deletions dominate), for each glycosylation factor differentially expressed (FDR<0.05) in a given cancer (each point represents a glycosylation factor and a specific cancer type). In red are genes predominantly amplified and with log2FC>0 in a given cancer (tumor versus normal). In blue are genes predominantly deleted and with log2FC<0.
The number of genes/cancer pairs in each quadrant is indicated. The names of a few genes in the diagonal are indicated (the cancer corresponding to those points are omitted for simplicity).

Supplementary Figure S6

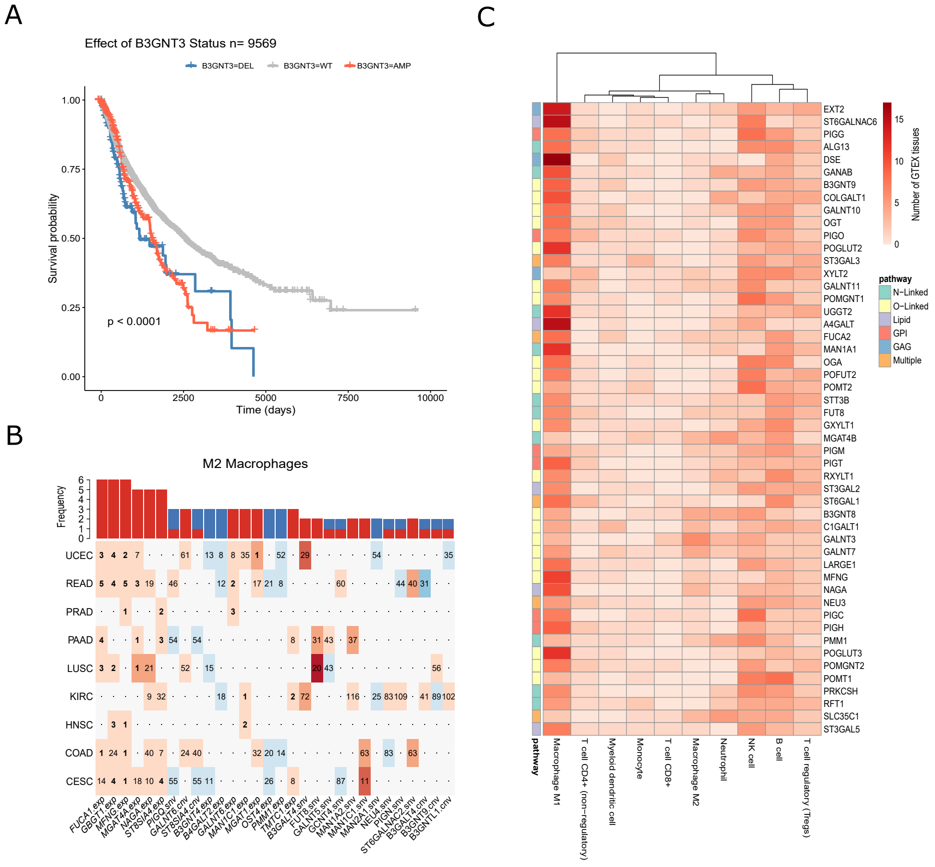

Supplementary Figure S6: Glycosylation Factors have prognostic value in cancer. **A)** Kaplan-Meier curves representing the effect on survival time of CNAs in the B3GNT3 gene (over all TCGA samples, n=9569). **B)** Heatmap representation of the significant coefficients for gene perturbation events in a lasso-regression model correlating events with estimated frequency of M2 macrophages (estimated by quantiseq) in TCGA samples. Red color indicates positive coefficients and blue color negative coefficients. Color intensity indicates value of the coefficient, with darker tones indicating higher values for the coefficient. Numbers indicate relative importance of the event in the regression model. Only cancers with an overall R>0.3 for the model are displayed. **C)** Heatmap representation of the number of GTEX tissues where we can find a significant (p<0.05) correlation between glycosylation factor gene expression and estimated frequency of immune cell populations (estimated using quantiseq). Only the top 50 genes with significant correlations in more tissues are displayed.
